# BAck iN the Game (BANG) – a smartphone application to help athletes return to sport following anterior cruciate ligament reconstruction: protocol for a multi-centre, randomised controlled trial

**DOI:** 10.1186/s12891-020-03508-7

**Published:** 2020-08-08

**Authors:** Clare L. Ardern, Joanna Kvist, on behalf of the BANG Trial Group, Clare Ardern, Joanna Kvist, Anne Fältström, Anders Stålman, Paul O’Halloran, Kate Webster, Nicholas Taylor

**Affiliations:** 1 Division of Physiotherapy, Karolinska Institute, Stockholm, Sweden; 2 Unit of Physiotherapy, Linköping University, Linköping, Sweden; 3 Sport and Exercise Medicine Research Centre, La Trobe University, Melbourne, Australia

**Keywords:** Return to play, Knee, Rehabilitation, Telemedicine, Mobile health

## Abstract

**Background:**

Sustaining injury is a common consequence of playing sport. At least one in every three recreational athletes with anterior cruciate ligament (ACL) reconstruction do not return to their preinjury sport following treatment. Psychological factors including confidence and fear of new injury exert large effects on returning to sport. The primary aim of this trial is to test whether a custom smartphone application delivering cognitive-behavioural therapy is effective for improving the number of people who return to their preinjury sport and level following ACL reconstruction.

**Methods:**

Participants scheduled for primary ACL reconstruction are recruited prior to surgery from one of six trial sites in Sweden. We aim to recruit 222 participants (111 in each group) for the BANG trial. Participants are randomly allocated to receive either usual rehabilitation care alone or usual rehabilitation care plus the Back in the Game smartphone application intervention. Back in the Game is a 24-week Internet-delivered programme, based on cognitive-behavioural therapy. The primary outcome is return to the preinjury sport and level at 12 months follow-up. The secondary outcomes assess physical activity participation, new knee injuries, psychological factors, quality of life and physical function. Physical activity participation and new injuries are self-reported every two weeks for 12 months, then every 4 weeks to 24 months follow-up. Psychological readiness to return to sport, knee self-efficacy, motivation to participate in leisure time physical activity, knee-related quality of life, and self-reported knee function are also assessed at 3, 6, 9, 12 and 24 months after surgery. A clinical assessment of strength, knee range of motion, effusion and hopping performance is completed by a blinded assessor at 12 months to assess physical function.

**Discussion:**

This protocol outlines how we plan to assess the efficacy of a custom smartphone application, delivering cognitive-behavioural therapy to address fear, confidence and recovery expectations, for improving return to sport following serious sports-related musculoskeletal injury. The BANG trial employs a pragmatic design to best reflect the reality of, and inform, clinical practice.

**Trial registration:**

ClinicalTrials.gov, NCT03959215. Registered 22 May 2019.

## Background

Regular participation in sport and active recreation is advocated by the World Health Organization as important for improving global physical activity levels [1, 2]. At least one in every three adults do not meet recommendations for the amount of physical activity required for health benefits [3]. Participating in vigorous activities (i.e. sport) may afford greater health benefits than moderate physical activity [4–6]. Therefore, the health benefits of participating in organised sport should not be underestimated. The dilemma is, ill-health – sustaining injury – is a common consequence of playing sport.

Based on register data of serious sports and active recreation injuries, at 1 year after injury, one in three people had leisure time physical activity levels that were below the minimum recommended for health benefits [7]. People who stopped being active were highly active in sport before their injury. It is unclear whether the injury caused the person to quit playing sport. Rehabilitation clinicians often encounter the athlete who has the physical capacity to participate in sport, but is afraid to participate.

Returning to sport is usually the primary concern of athletes following injury. Yet after serious injury, like anterior cruciate ligament (ACL) tear, up to half of athletes do not return to competitive sport [8]. The transition through rehabilitation and back to sport can be difficult for injured athletes as they experience concerns about their ability to perform at the same level as before the injury, and anxiety about sustaining a new injury [9]. Adding to the challenge, is that (i) there is no systematic approach to addressing psychological concerns during sports injury rehabilitation, (ii) rehabilitation clinicians often feel ill-equipped to provide effective psychological support, [10] and (iii) during the time the athletes are returning to sport, they typically lack the support of a rehabilitation clinician, having been discharged from rehabilitation months earlier.

A self-directed, stand-alone intervention, serving as a complement to rehabilitation might be an effective way to overcome some of the barriers to delivering effective psychological support to athletes during, and after, rehabilitation. eHealth technology facilitates low-cost, on-demand delivery of psychological support to injured athletes [11]. Because smartphones are a ubiquitous part of daily life, they are an attractive platform from which to deliver evidence-based strategies for improving confidence to return to sport that athletes can access anywhere, and at any time – psychological support in the athlete’s pocket.

### Aims and hypothesis

The primary aim of this randomised controlled trial is to test whether Internet-delivered cognitive-behavioural therapy to address confidence for returning to sport (Back in the Game app), is effective for improving the number of people who return to their preinjury sport and level following ACL reconstruction.

The secondary aims are to assess the effect of the Back in the Game app on participation in sport, incidence of new knee injuries, psychological readiness to return to sport, knee self-efficacy, motivation to return to sport, knee-related quality of life, and self-reported knee function.

Our hypothesis is that more patients who use the app in addition to receiving usual rehabilitation care following ACL reconstruction will return to their preinjury sport and level than patients who receive usual care alone.

## Methods

**Fig. 1:**
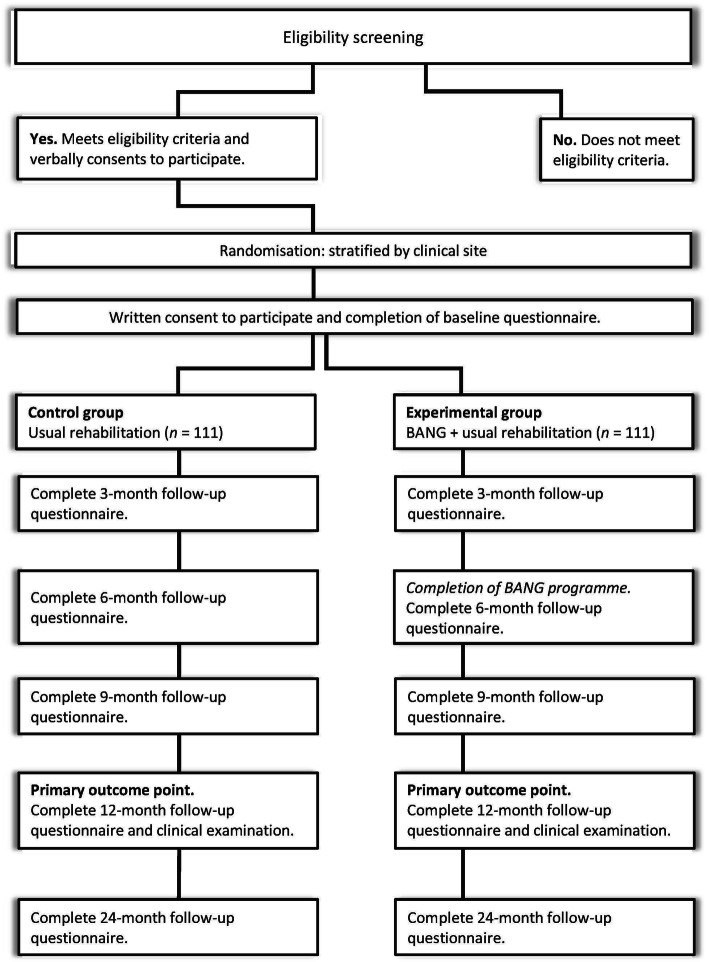
Flow of participants through the trial. Fortnightly physical activity and injury registration in the first year of follow-up, and monthly physical activity and injury registration in the second year of follow-up are not shown

The BANG trial is a parallel-group, two-arm, superiority trial. Participants with ACL reconstruction will be recruited from one of six clinical sites and randomly allocated (in blocks of 6 with a 1:1 allocation, stratified for site) to receive either usual rehabilitation care alone (control group) or usual rehabilitation care plus the Back in the Game smartphone application (experimental group) (Fig. 1). The primary outcome is return to the preinjury sport and level at 12 months after surgery.

### Study setting

Participants are recruited from three metropolitan private hospitals, one University hospital and three regional public hospitals in southern and western Sweden.

### Recruitment process

The clinic co-ordinator at each site completes eligibility screening of patients who have been scheduled by their treating orthopaedic surgeon for ACL reconstruction. Patients who meet the eligibility criteria are provided written information about the trial. Approximately 5 days later, patients are asked if they wish to consent to participate and be contacted by the trial co-ordinator.

The trial co-ordinator contacts participants via email and short message service (SMS) with information about how to download the app and create an account. A second round of consent to participate in the study occurs at this point – participants must complete electronic informed consent before their app account is registered. Recruitment commenced in June 2019 and is anticipated to conclude in December 2020.

#### Recruitment rate and strategies to achieve adequate participant enrolment

Approximately 4000 ACL reconstructions are performed annually in Sweden; the trial sites perform approximately one third of these surgeries [12]. Patients aged between 16 and 30 years comprise approximately 60% of all patients receiving ACL reconstruction in Sweden [12]. Based on our previous experience, we expect approximately 20 to 40% of patients to be eligible to participate in the BANG trial (typical clinical population varies at the clinical sites – we expect the metropolitan sites will have a higher proportion of potentially eligible patients). We expect approximately 50% of eligible patients to consent to participate in the trial.

We aim to recruit 222 participants (111 in each group) for the BANG trial. Recruitment progress at each trial site is formally reviewed every 6 months to judge the likelihood of completing recruitment according to the planned timeline. If recruitment is slower than anticipated, we will consider approaching additional clinical sites to assist with participant recruitment.

### Eligibility criteria

Participants who meet the following criteria will be included in the trial:

- Age 15 years to 30 years at the time of ACL injury
- Unilateral primary ACL rupture (diagnosed by clinical examination and/or MRI)
- Time between injury and ACL reconstruction not more than 12 months
- Playing contact pivoting or non-contact pivoting sport at least twice per week prior to ACL injury
- Intend to return to sport following ACL reconstruction
- Normal/healthy contralateral knee
- Fluent in written and spoken Swedish language

Participants with the following will be excluded from the trial:

- Medial collateral ligament or lateral collateral ligament injury requiring surgery
- Posterior cruciate ligament injury
- Meniscus injury and/or treatment requiring alteration to usual rehabilitation care
- Articular cartilage injury and/or treatment requiring alteration to usual rehabilitation care
- Previous ACL injury to either knee
- Injury to either lower limb that required medical care during the 12 months prior to index ACL injury
- Other injury or illness that could affect knee rehabilitation
- Taking medication for mental health problems

We recruit young, pivoting sport athletes who desire to return to sport, because the intervention is tailored to address the mental challenges these athletes typically report [13–17]. Participants must have sufficient Swedish language comprehension because the intervention is only available in Swedish. Previous experience of ACL injury, [18] and social and contextual factors, including age and desire to return to sport, [8, 19, 20] may also influence returning to sport. Our eligibility criteria will help minimise the confounding effect of injury history, age and desire to return to sport on the trial results.

### Interventions

The Back in the Game app and usual rehabilitation care are summarised according to the Template for Intervention Description and Replication (TIDieR) Checklist [21] (Table 1). All participants complete post-operative rehabilitation as normal. As part of usual rehabilitation care in Sweden, patients typically receive generic written information about knee injury and surgery, recovery in the early post-operative period, return to sport, and injury prevention. A summary of the typical written information patients receive from their treating clinician is delivered to both groups via the smartphone application. The Back in the Game app intervention delivers additional, tailored information and cognitive-behavioural therapy exercises to complement post-operative rehabilitation.

**Table 1:** Overview of usual rehabilitation care (control) and the Back in the Game intervention (experimental). All participants receive usual post-operative rehabilitation care

TIDieR item	Control	Experimental
**Name**	Usual post-operative rehabilitation care	Back in the Game plus usual post-operative rehabilitation care
**Why**	Usual care reflects the real-world clinical context. Rehabilitation helps patients recover from surgery, gradually regain knee function, and prepare to return to sport.	Psychological factors such as confidence and anxiety about new injury, have strong influences on returning to sport after serious knee injury. Rationale: supporting psychological readiness to return to sport, in addition to usual rehabilitation, will help athletes transition back to their sport. A self-directed approach will help target the specific challenges encountered by the individual.
**What (materials)**	Strength training equipment (e.g. free weights, machine weights, resistance bands, suspension cables), balance training equipment (e.g. BOSU ball) and aerobic training equipment (e.g. treadmill, stationary bicycle) as available in the usual care setting.	All content is provided on-demand through the Back in the Game smartphone application (see https://vimeo.com/345486299/6bba07cc11 for an overview [in Swedish]).
**What (procedures)**	Usual rehabilitation care for pivoting sports athletes typically comprises 4 phases [22]: 1. Acute phase aimed at reducing pain and swelling, improving knee movement, and recovering performance of activities of daily living (e.g. walking without aids). 2. Intermediate phase aimed at progressing muscle strength sport-specific tasks 3. Late phase 4. Injury prevention phase The treating clinician and the patient collaborate to decide on the specific therapies and exercises, and the number of face-to-face, home-based and gymnasium-based treatment/training sessions required.	Users receive a notification at least every 2 weeks to complete tasks relevant to their stage of rehabilitation. The intervention is designed to be complementary to patients’ rehabilitation progression. The 24-week programme is based on cognitive-behavioural therapy principles, and comprises 7 modules: 1. Goal setting 2. Confidence for recovery 3. Confidence for return to sport 4. Confidence for performance 5. Confidence to stay injury-free 6. Support to handle thoughts and emotions related to recovery and return to sport 7. Education about knee injury, recovery, return to sport, and safe sports participation
**Who**	Physiotherapist plus patient-directed home and/or gymnasium-based sessions	Patient-directed via custom application
**How**	Typically, individual face-to-face treatment sessions combined with independent sessions at home and/or gymnasium. Some clinicians may provide group rehabilitation sessions.	Internet-delivered (smartphone or desktop application)
**Where**	Swedish outpatient rehabilitation clinic (either public/primary care or private setting) plus home and/or gymnasium-based programme at a convenient location for the participant.	Users access the intervention on-demand via custom application
**When**	The duration of rehabilitation is highly varied. Post-operative rehabilitation programmes typically run for at least 6 months, and usually cease by 12 months.	A 24-week programme commencing one week following ACL reconstruction (i.e. in the first post-operative week). Users continue to have access in ‘read-only’ mode from the end of the active intervention period (24 weeks) up to 12 months following ACL reconstruction.
**How much**	Highly variable. Clinical practice guidelines recommend at least 3–4 sessions per week. In the early post-operative phase, rehabilitation sessions may be short duration and more frequent. In the late phase, rehabilitation sessions may be longer duration (minimum 40 min) and less frequent.	Minimum 30 min recommended training per week over the 24-week programme. Users receive up to 3 SMS reminders per new task.
**Tailoring**	The treating clinician directs rehabilitation content to focus on specific impairments or functional limitations as appropriate for the patient’s daily living and sport demands. We expect the exercises chosen will be highly variable but will cover the 4 broad rehabilitation phases.	Users choose the cognitive-behavioural therapy task they would like to practice each week from a task menu. Intervention modules are tailored to the progression of rehabilitation (e.g. focus on confidence in recovery during the early rehabilitation phase and confidence to perform well in sport during the late rehabilitation phase).
**How well**	Participants record how many sessions per week they attend of face-to-face rehabilitation, and how many home-based and gymnasium-based rehabilitation sessions they complete.	The research team will track use usage statistics.

TIDieR, template for intervention description and replication; ACL, anterior cruciate ligament

#### Back in the Game app

**Fig. 2:**
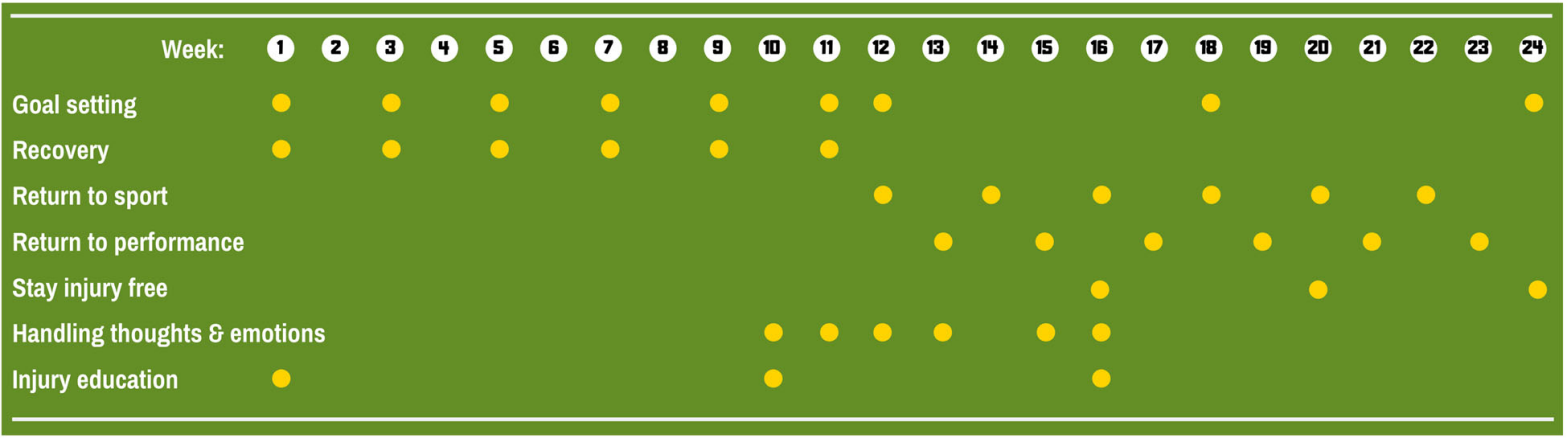
Summary of the Back in the Game intervention. Each row represents a self-directed module. Each dot represents how frequently tasks are delivered to the user

A 24-week programme (Fig. 2) based on cognitive-behavioural therapy principles, and designed to deliver on-demand psychological support targeting the psychological barriers to athletes returning to sport. There are 7 self-directed modules to build confidence to return to sport that are designed to progress in complexity and mirror progress in post-operative rehabilitation.

Each module includes a progression of different tasks: short-term and return to sport goal setting, visualisation, relaxation/meditation, exercises to reduce fear, watching interviews with previously injured athletes (plus self-reflection questions), reading information about managing pain and the thoughts and emotions arising during rehabilitation, and developing an action plan for staying confident to return to sport. Users receive notifications to complete new tasks at least each fortnight (Fig. 2).

At the conclusion of the active 24-week intervention period, users have access to all modules and tasks in ‘read-only’ mode; users will not be sent notifications to engage with the intervention after 24 weeks.

#### Usual rehabilitation

Following ACL reconstruction, as per routine clinical practice, all participants are referred to outpatient physiotherapy. Overall treatment aims, content and progression of rehabilitation will follow current clinical guidelines [23]. The choice of specific therapies, exercises and the number of treatment sessions needed to achieve the required treatment aims is at the clinical discretion of the treating physiotherapist.

### Outcomes

The primary outcome is the proportion of participants who return to their preinjury sport *and* participation level at 12 months. The secondary outcomes (Table 2, Additional file 1) are measured at clinically-relevant times, and reflect a biopsychosocial approach to outcome evaluation: patient- and clinician-reported knee function, sports participation and new knee injuries for the biological aspect, psychological readiness to return to sport and self-efficacy for the psychological aspect, adherence to the intervention and/or rehabilitation, and a range of baseline characteristics (Table 3, Additional file 1) for the social aspect.

**Table 2:** Summary of secondary outcomes

Measurement variable	Aggregation method	Measurement time point(s)
Sports participation	Number of minutes playing (i) contact, pivoting sports, (ii) non-contact, pivoting sports, (iii) non-pivoting sports	Every 2 weeks to 12-months follow-up; every month from 12 to 24 months follow-up
New knee injuries	Proportion of participants who report a new (i) ACL injury, (ii) meniscus injury, (iii) other knee injury or problem	Every 2 weeks to 12-months follow-up; every month from 12 to 24 months follow-up
Psychological readiness to return to sport	Mean or median ACL-Return to Sport after Injury scale [24] score	3, 6, 9, 12, 24 months
Knee-related self-efficacy	Mean or median Knee Self-Efficacy Scale [25] future domain score	12, 24 months
Motivation to return to sport [26]	Median	3, 6, 9 months
Knee-related quality of life	Mean or median ACL-Quality of Life scale [27] score	12, 24 months
Self-reported knee function	Mean or median International Knee Documentation Committee subjective knee form [28] score	6, 12, 24 months
Self-reported knee function	Mean or median Single Assessment Numeric Evaluation [29]	Every 2 weeks to 12-months follow-up; every month from 12 to 24 months follow-up
Functional knee stability	Frequency of giving way episodes	12, 24 months
Knee effusion	Proportion of participants with stroke test [30] score: 0, trace, 1+, 2+, 3+	12 months
Hopping performance [31, 32]	Mean or median limb symmetry index	12 months
Quadriceps and hamstrings strength	Mean or median limb symmetry index	12 months
Adherence to rehabilitation	Number of sessions completed	Every 2 weeks while completing rehabilitation
Adherence to BANG intervention	Number of completed video and audio sessions	6 months

Limb symmetry index is calculated using the formula: $\frac{involved\ limb}{uninvolved\ limb}\times 100$; ACL, anterior cruciate ligament

**Table 3:** Baseline participant characteristics

Variable	Aggregation
Time from injury to surgery	median (IQR)
Age at injury	mean (SD)
Sex	
Female	n (%)
Male	n (%)
Primary occupation	
Student	n (%)
Desk work	n (%)
Manual work	n (%)
Heavy manual work (e.g. construction)	n (%)
Unemployed	n (%)
Preinjury sports participation	
Contact, pivoting sport	n (%)
Non-contact, pivoting sport	n (%)
Return to sport goal	
Return to same sport	n (%)
Return to different sport	n (%)
Return to sport expectations	
Within 1 month	n (%)
Within 6 months	n (%)
Within 12 months	n (%)
After 12 months	n (%)
IKDC subjective knee form [28] score	mean (SD)
General Self-efficacy Scale [33] score	mean (SD)
Knee Self-Efficacy Scale [25] *Future domain*	mean (SD)
Hospital Anxiety & Depression Scale [34] score	mean (SD)

*IQR* interquartile range, *SD* standard deviation, *IKDC* International Knee Documentation Committee

#### Primary outcome measure

For the primary outcome, return to sport is determined based on a composite of answers to 4 questions (Additional file 1), which we have refined based on our previous research [19, 35, 36].

To be classified as returned to the preinjury sport *and* level (i.e. *yes* for the primary outcome), participants must have answered *I have returned to the same sport as before injury* to question 1, AND *I have returned to full training and modified competition* or *I have returned to full training and full competition* to question 2, AND selected the same sport as they played preinjury at question 3, AND selected the same level as they played before injury at question 4 (to ensure accuracy, we cross-check answers to question 3 and question 4 at 12 months, against the baseline data).

#### Secondary outcome measures: *sports participation*

Participants self-report the activity or activities (participants can register up to 3 separate activities) they participated in during the registration period (preceding two weeks). Participants select an activity from a list of 19 sports; there is also an option to specify an unlisted activity. Participants report the number of minutes they participated in each activity during the registration period, and the total number of physical activity sessions including, but not limited to knee rehabilitation sessions, active recreation, training/practice and competition.

#### Secondary outcome measures: *new knee injuries*

Participants self-report any new knee problems that have occurred during the registration period (preceding two weeks). We use an ‘all complaints’ definition of injury [37]. Participants describe the problem, and any diagnosis and treatment received (including the clinician and/or institution where diagnosis and/or treatment was provided).

#### Secondary outcome measures: *patient-reported outcomes*

To assess *psychological readiness to return to sport*, we use the condition-specific ACL-Return to Sport after Injury (ACL-RSI) scale – a 12-item scale, scored from 1 to 10; higher scores indicate greater psychological readiness to return to sport [38].

To assess *self-efficacy*, we use the *future* domain of the Knee Self-Efficacy Scale (K-SES) [25]. The domain comprises 4 questions to assess self-efficacy related to future knee function: (1) how certain are you that you can return to the same physical activity level as before the injury?, (2) how certain are you that you would not suffer any new injuries to your knee?, (3) how certain are you that your knee would not ‘break’?, (4) how certain are you that your knee will not get worse than before surgery? Each question is scored on a 0 to 10 scale. The domain score is the mean of responses to the 4 questions, and higher scores represent stronger self-efficacy.

For *motivation to return to sport*, we ask 3 questions, all measured on a 1 to 10 scale [26]: (1) how important is it for you to return to the same sport or recreation activity as before your knee injury?, (2) do you think it is possible for you to return to the same sport or recreation activity as before your knee injury?, (3) how much time and effort are you willing to invest to return to the same sport or recreation activity as before your knee injury?

To assess *quality of life*, we use the ACL-Quality of Life (ACL-QoL) scale [27] – a 32-item questionnaire, scored from 0 to 100; higher scores indicate greater knee-related quality of life. The ACL-QoL is the only condition-specific measure of quality of life available for people with ACL injury.

For *patient-reported knee function*, we use the Single Assessment Numeric Evaluation (SANE) [29] (“On a scale from 0-100 where 100 represents the best, what number would you give your knee today?”) and the International Knee Documentation Committee (IKDC) subjective knee form, [28] a 19-item condition-specific measure. The SANE and the IKDC subjective knee form measures are both scored out of 100 points, with a higher score indicating superior self-reported knee function.

For *functional knee stability*, we use ask participants to report (i) how many times (if any) their knee has given way after surgery, and (ii) how often their knee gives way.

#### Secondary outcome measures: clinician-measured knee function

For quadriceps and hamstrings strength, we use an isokinetic dynamometer to record concentric peak torque in a seated position. We measure 5 repetitions at 60°/sec and 15 repetitions at 180°/sec.

**Fig. 3:**
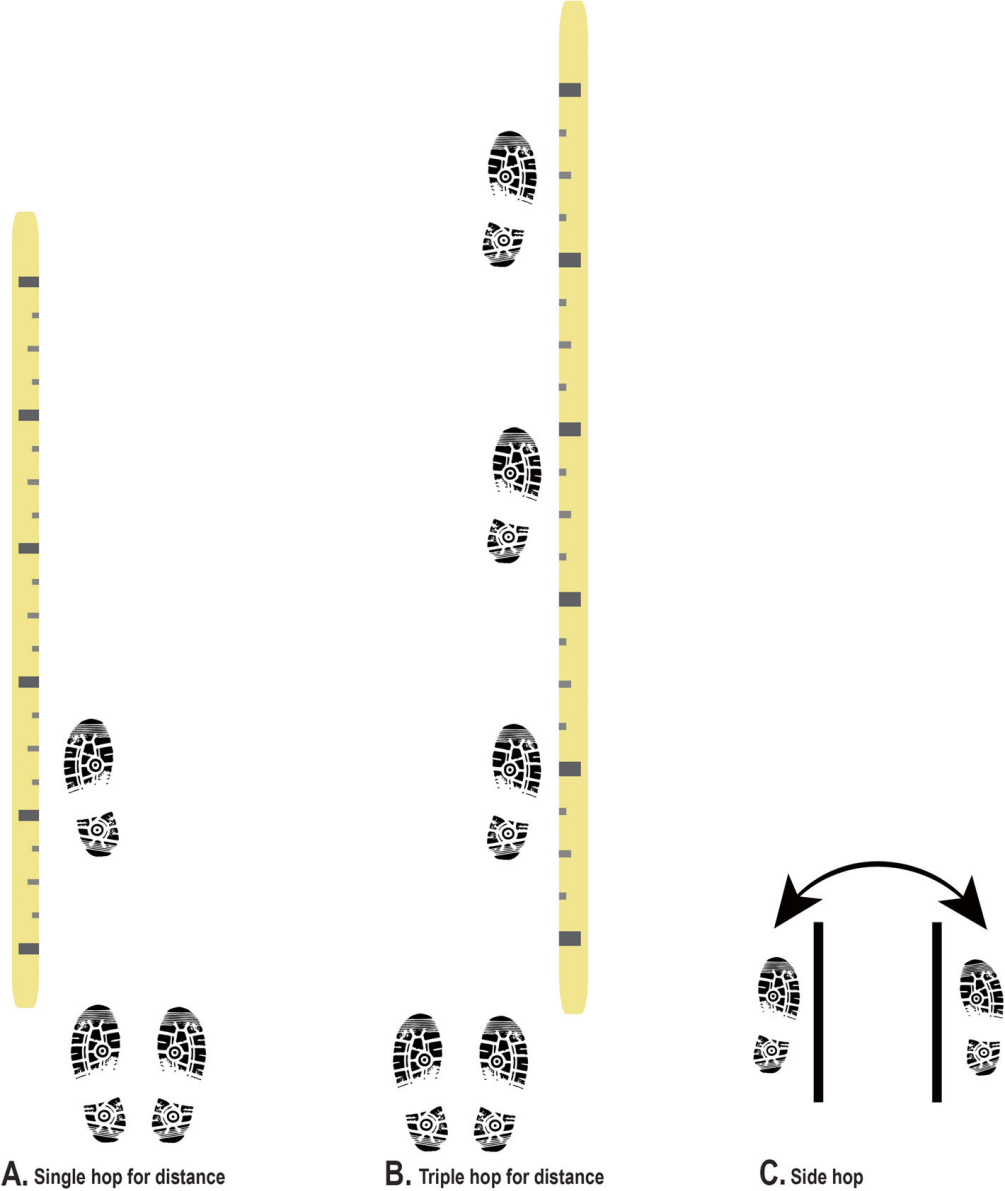
Visual summary of hopping performance tests

For hopping performance, we use three different tests (Fig. 3) [31, 32]. The single hop for distance test is the maximum distance the person can hop from a stationary starting position. The triple hop for distance test is the maximum distance the person can hop with three successive hops from a stationary starting position. The side hop test is the number of hops the person can complete side-to-side (minimum width 40 cm), in a 30-s period.

For knee effusion, we use the stroke test [30] to grade the amount of knee effusion as no fluid wave, trace (small fluid wave), 1+ (large fluid wave), 2+ (fluid wave spontaneously returns to the medial aspect of the knee) or 3+ (excess fluid that cannot be moved away from the medial aspect of the knee).

#### Secondary outcomes measures: *adherence*

We will assess adherence to the Back in the Game intervention during the 24-week intervention period by counting page views for different types of content (e.g. Vimeo analytics for video content, SoundCloud analytics for audio content). We will assess adherence to rehabilitation every two weeks by asking participants to report the number of supervised physiotherapy sessions, number of home-based exercise sessions and the number of gymnasium-based exercises sessions completed in the previous two weeks.

### Assignment of interventions: allocation—sequence generation

We use a cluster-randomisation strategy. An independent statistician created a computer-generated randomisation schedule for each clinical site (hospitals that belong to the same clinical network are classified as one clinical site for randomisation) using computer-generated random numbers in blocks of 6. We use block randomisation because we expect faster recruitment rates at the high-volume private metropolitan clinical centres.

### Allocation—concealment mechanism

Allocation is concealed in sequentially numbered, opaque, sealed envelopes. When the central study co-ordinator is notified that a participant has consented to participate in the study, she opens the next envelope in the sequence. Allocation is managed at a location remote to the clinical sites.

### Blinding

Outcome assessors and data analysts are blinded after assignment to intervention. Because the Back in the Game app is a self-directed intervention and designed as a standalone to usual rehabilitation care, there are no additional care givers who should be blind to participant allocation.

Baseline data are collected via the smartphone app, prior to allocation and the intervention commencing. Before they accept participation, all participants are informed that (1) they will receive content guiding them about returning to sport via the smartphone app, and (2) that depending on which group they are allocated to, they will receive more or less content. We do not provide further description of the intervention prior to participants consenting to participate, to avoid inducing participation. Participants are informed that if they agree to be in the trial, there is a 50–50 chance of receiving more than the usual amount of content about return to sport provided during post-operative rehabilitation. Participants are not informed of their group allocation, but we cannot assume they remain blinded to group allocation.

When they return for clinical assessment at 12 months follow-up, we will ask participants not to discuss the amount and content of information they received during post-operative rehabilitation with the outcome assessor. We plan multiple strategies to minimise the chance of a participant inadvertently disclosing allocation to the outcome assessor including sending the participant a reminder SMS message one day prior to the follow-up visit.

### Informed consent

The clinical co-ordinator provides a description of the trial to patients (written and verbal information) and discusses the information provided. After the eligibility screening process, patients can verbally consent to participate in the trial and be contacted by the trial co-ordinator. Patients provide electronic informed consent through the software platform used for all study data collection.

### Sample size calculation

There are no trials of psychological support interventions aimed to help athletes return to sport following sports injury, so it is difficult to accurately estimate the expected effect size for our trial. We calculated the same size required to detect a between-group difference in return to preinjury sport rate of 20% at 1 year. We assumed a base return to sport rate of 33%, [39] a two-sided alpha of 0.05, beta of 0.2, and a drop-out rate of 15%. The required sample size is 222 participants (111 in each group).

### Data collection

All participants download the Back in the Game app, which is how we deliver the intervention, and administer all research data collection. Participants in the experimental group have access to a separate part of the app that only goes ‘live’ when the intervention commences. Participants in the control group cannot see the ‘hidden’ intervention part of the app.

Baseline measurements are collected prior to allocation. Participants complete the baseline questionnaire in the week prior to ACL reconstruction surgery, providing demographic information, sports participation, and a baseline measure of self-reported knee function, self-efficacy, and anxiety and depression (Table 3). The Internet-delivered intervention commences in the first post-operative week (Table 4). Permissions to access the intervention can only be assigned at the time the participant sets up their own profile in the app and provides informed consent.

**Table 4:**
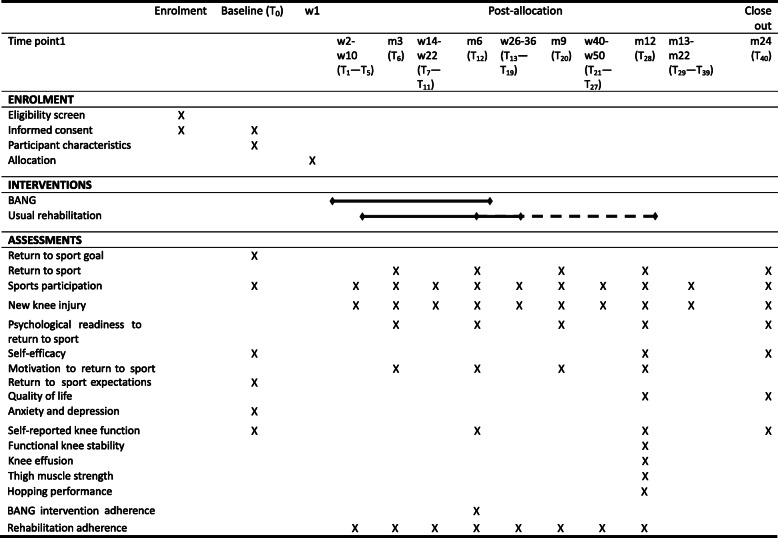
Overview of key time points in the BANG trial

w, week; m, month; T, time point (the superscript number is cumulative across the trial data collection and denotes how many times the outcome is measured during the data collection window, i.e. T_1_ – T_5_ denotes five data collection times points, T_6_ denotes one data collection time point); solid line denotes duration of BANG intervention and expected duration of usual rehabilitation, dashed line denotes time when some participants might be completing usual care rehabilitation because rehabilitation duration is variable, but it is uncommon for rehabilitation to continue beyond 12 months post-operative

For all patient-reported outcomes, participants will receive up to 3 notifications to complete the questionnaire (1 invitation and up to 2 reminders). Administration of all patient-reported outcomes is managed by the trial co-ordinator. The clinical assessment will be performed at each clinical site, at 12 months post-operative. Measurements will be recorded on an electronic template (Additional file 1) for each participant.

#### Clinician-measured knee function

An independent, blinded assessor will conduct the tests. Participants will complete a standardised warm-up for at least 8 min (either stationary bicycle or treadmill run at rating of perceived exertion of between 12 and 16 (around “somewhat hard”) on the Borg scale [40]) prior to the strength and hop tests. For each strength and hop test, participants will complete at least 1 familiarisation trial on each leg, until they feel comfortable to perform the test. The number of familiarisation trials is at the discretion of the assessor. Strength tests will always be performed before the hop tests because of the maximal effort nature of the test. Participants will have a minimum 5-min rest between completing the strength tests and commencing the hop tests. All tests are completed with shoes on. If participants are unable to complete any one or more of the strength or hop tests, the assessor will record the result as not attempted and the reason why.

Each assessor will be trained in standardised measurement of strength and hop performance. Each clinical site has an isokinetic dynamometer for strength testing. Because the strength and hopping tests are performance tests, assessors will provide vigorous feedback to elicit a maximum test result. Each limb is tested separately; the uninjured limb is always tested first. The hop tests are performed with the participant’s hands clasped behind his or her back.

For the strength tests, the starting position is approximately 110° knee flexion. Participants are instructed to repeatedly straighten and bend their knee as hard and as fast as possible, against the resistance of the dynamometer.

For the single and triple hop for distance, the instruction is to stand on the test leg, then to hop as far forward as possible, taking off and landing on the same foot, and with a controlled, balanced landing. On landing, the foot must remain in the same place – no additional hops allowed – for up to 3 s. The participant will complete two trials on the uninjured leg and two trials on the injured leg, with a minimum 20 s rest between trials. The longest distance hopped will be recorded for both limbs.

For the side hop test, the participant stands on the test leg and hops from side-to-side, over two parallel strips of tape placed 40 cm apart, as many times as possible in 30 s. If the foot touches the either strip of tape, the hop is invalid and not counted. Each trial will be videorecorded to assist the assessor to count valid hops. There is a minimum 90 s rest between completing the trial on the uninjured leg and completing the trial on the uninjured leg. Participants complete one trial on each leg. If > 25% of the hops are invalid, the participant will complete a second trial after at least 3 min rest.

### Data management plan

The trial co-ordinator is responsible for administering all research data collection. The trial co-ordinator or research assistant will monitor questionnaire responses and attempt to contact participants via text message if there are missing data. The blinded assessor who completes the 12-month clinical assessment will check the data collection template prior to sending the data to the trial co-ordinator, to ensure there are no missing data. Researchers from the BANG Trial group will check the coded database for missing, implausible and inconsistent data prior to analysis.

The trial co-ordinator and principal investigators will oversee data management and have access to the full dataset. All BANG Trial group researchers will have access to coded data, on reasonable written request. The trial will comply with Swedish data protection law.

#### Monitoring

A data monitoring committee (DMC) will be established according to the DAMOCLES Group recommendations (see Additional file 1 for DMC charter) [41]. The DMC will be independent of the trial investigators. During the period of trial recruitment, interim analyses will be conducted by the trial statistician and provided to the DMC. The DMC will be free to request any other analyses the committee requires, and these analyses will be provided in the strictest confidence.

The frequency of interim analyses will be determined by the Chair of the DMC. However, we anticipate that there might be one interim analysis and one final analysis. The DMC will comprise at least 3 independent committee members (including at least one clinician and one researcher). One or more trial investigators may attend the DMC meetings, but the decision-making is limited to the independent DMC members.

An interim analysis of adverse events (using generalised estimating equations) may be performed when 50% of participants have been randomised and have completed the 12-month follow-up. The interim analysis will be performed by the trial statistician, blinded to treatment allocation, who will report to the DMC. The DMC will have access to the unblinded data and will discuss the results of the interim analysis. The DMC will recommend whether the trial should continue.

#### Harms

Participants report any adverse events – an untoward physical or psychological occurrence with or without an expected causal relationship to the intervention (e.g. deep vein thrombosis, embolism, superficial or deep infection to the index knee, psychological distress, increase in knee symptoms, ligament sprain, muscle strain, meniscus tear) to the trial co-ordinator via electronic questionnaire (open-ended question sent every 2 weeks during the first year of follow-up; we expect usual care rehabilitation to have a duration of approximately 9 months). A serious adverse event for this trial is any untoward physical or psychological occurrence that the trial investigators believe is causally related to the intervention *and* is any of the following: life-threatening, associated with permanent and/or severe disability, associated with prolonged hospitalisation. Serious adverse events will be reported to the regional ethics review board.

#### Missing data

If required, we will use multiple imputation to account for missing data in analyses that cannot handle occasional missing values [42]. If there are missing data for the primary outcome, we will either: (i) substitute from the fortnightly sports participation data, or (ii) use multiple imputation if there are > 15% missing outcome data. If there are missing data for the secondary outcomes, we will use multiple imputation if there are > 15% missing outcome data.

#### Reclassifying variables

For new knee injuries, we plan to classify the collected data into three dichotomous variables for analysis: (i) new knee problem (based on all complaints), (ii) new ACL injury (either knee), (iii) new knee injury treated with surgery. The trial co-ordinator will check all injury registration reports as they are completed and returned by participants. If additional information (e.g. injury diagnosis and treatment) is required, a blinded researcher will contact the participant by phone or text message, and/or review the participant’s medical record (Additional file 1).

#### Data security

All data are managed according to the European Union General Data Protection Regulation. A password-protected electronic record of all trial activities will be maintained according to University requirements. Patient-reported data are collected electronically, coded according to the participant’s unique study identification number, and stored electronically on a password-protected University server. Clinical follow-up data are collected using an electronic template, coded according to the participant’s unique study identification number, and stored electronically on a password-protected University server.

Identified data (including personal information; the coding list will be stored separate to the identified data) will be stored at the co-ordinating University for 10 years after completion of the study. Coded data and statistical code for generating results will be deposited on the Swedish National Data Service server within 6 months of study completion, and will be available indefinitely.

#### Data sharing

We plan to share coded individual participant data for the primary and secondary outcomes, and statistical code, with researchers whose proposed use has been approved by an independent review committee. Individual participant data will only be shared for the purpose of individual participant data meta-analysis. Data must be requested through the Swedish National Data Service (http://snd.gu.se).

### Statistical analysis plan

For the main analysis, we will use the intention-to-treat principle. An independent statistician, blinded to group allocation, will perform the analyses using the coded database. The criterion for statistical significance will be < 0.05.

To assess baseline comparability of treatment groups, we will compare baseline characteristics (Table 3) using generalised estimating equations. If there are major imbalances in baseline characteristics, we will attempt to rebalance the groups using multivariable regression modelling.

#### Primary analysis

To address the primary aim, we will compare the rate of returning to the preinjury sport and level at 12 months between the treatment groups. We will calculate the odds ratio and 95% confidence interval using generalised estimating equations. Group and clinical site will be included as fixed factors in the model.

#### Planned secondary analyses: time-varying analyses

We will compare psychological readiness to return to sport (ACL-RSI), motivation to return to sport, self-reported knee function (SANE and IKDC), sports participation and new knee injury between treatment groups using generalised estimating equations. Group, clinical site, and assessment time point will be included as fixed factors. If baseline values were collected, we will also include the baseline value in the relevant model as a covariate. For continuous variables, we will calculate mean difference and 95% confidence intervals. For dichotomous variables, we will calculate the odds ratio and 95% confidence intervals.

#### Planned secondary analyses: cross sectional analyses

For self-efficacy (K-SES) and quality of life (ACL-QoL), we will use generalised estimating equations for comparisons at 12 months (model 1) and 24 months (model 2) between treatment groups. Group and current participation in preinjury sport will be included as fixed factors. We will calculate mean difference and 95% confidence intervals. In model 2, we will include the 12-month values as a covariate.

For adherence, we will compare the median number of rehabilitation sessions completed per person (i) with the physiotherapist, (ii) at home, (iii) at a gymnasium between the treatment groups using generalised estimating equations.

## Dissemination

We aim to develop a multifaceted approach to dissemination, targeting the key end users of the trial results: people with ACL injury, coaches and sports clubs, orthopaedics, sports medicine and rehabilitation clinicians, and sports medicine/injury researchers.

The results of the trial will be disseminated to patients and the public via blog, podcast and the University website. We plan to collaborate with athletes, coaches and sports clubs to disseminate information to those most likely to be affected by ACL injury.

We plan to provide public lectures through our sports medicine networks to disseminate the trial response to clinicians who work with athletes. We will also apply to present the trial results at key international sports medicine conferences.

Written reports of the research findings will follow the Consolidated Standards of Reporting Trials (CONSORT) Statement guidelines. Research publication authorship will be determined according to the International Committee of Medical Journal Editors guidelines; other contributors who do not meet the criteria for authorship will be acknowledged. All decisions about analysis, dissemination and publication will be made by the researchers – the funders will not be involved. We do not intend to employ a professional medical writer.

## Patient and public involvement in the research

There are two aims of patient and public partnership in this trial: (1) to guide the content, delivery and dissemination of the Internet-delivered intervention, and (2) to inform the research team about the burden of the intervention and participation in the trial.

Athletes with experience of ACL reconstruction and return to sport contributed to content development for the Internet-delivered intervention. We conducted a feasibility study focusing on acceptability, demand, practicality and integration. Eight patient-partners who had had ACL reconstruction within the preceding 8 weeks were recruited via our clinical contacts in a major metropolitan hospital and a university hospital in Sweden. Patient-partners used the Back in the Game app over a 10-week period and provided feedback (up to 3 times, via semi-structured interview) about the intervention content and delivery, the burden of the intervention, and time required to participate in the trial. Based on patient-partner feedback, we added additional content to help users get the most out of the intervention.

The main research question and choice of primary and secondary outcomes was informed by clinical and research experience. Return to sport is a primary reason why patients choose to have ACL reconstruction, [43] psychological factors are the principle barriers to returning to sport, [9] and return to sport is a patient-important outcome [44].

Patient-partners were not involved in trial design, recruitment to, or conduct of the trial. At the conclusion of the trial, we plan to collaborate with athletes, coaches and sports clubs to disseminate the results. Our patient and public partners will have input into the decision about what results to share, when and in what format.

## Discussion

This protocol outlines how we plan to conduct the BANG trial to assess the efficacy of an Internet-delivered intervention, delivering cognitive-behavioural therapy to address psychological factors including fear, confidence and recovery expectations (Back in the Game app), for improving the return to sport rate following ACL reconstruction. Promoting life-long participation in sport and active recreation is a critical public health priority, especially given physical inactivity is one of the top-five risk factors for noncommunicable disease [1].

Sustaining injury is often a consequence of playing sport. Some injuries, like a minor ankle sprain, might result in minimal time lost from sport – the athlete quickly resumes normal training, and continues to participate as before her injury. Other injuries require an extended period of the sidelines of sport. ACL rupture is one of the most common sports-related knee injuries, and injured athletes regularly spend at least 12 months on the sidelines of their sport. Some athletes may never regain their enjoyment for participating in sport after serious injury; many fear sustaining the injury again if they go back to their sport [9]. For some athletes, serious injury may be the catalyst for retiring from sport.

### A clinically-relevant and applicable intervention

A main concern for newly injured athletes is whether they will return to their previous sports participation. Many do not return, despite return to sport being a key goal early after injury, and a key focus of rehabilitation. The on-demand, smartphone-delivered intervention being studied in the BANG trial reflects a paradigm-shift to rehabilitation that comprehensively addresses the key barriers to athletes’ returning to sport: low confidence and high fear of reinjury [35].

Lack of confidence is a serious barrier to otherwise healthy, young athletes reaching their return to sport goal after ACL injury. Structured psychological support may be beneficial, yet most athletes do not access sport psychology for varied reasons that may include cost, stigma and geographic remoteness. The self-guided, on-demand delivery mode of the Back in the Game app is discreet, no cost to users, and available to access at a time that fits with the user’s needs and lifestyle.

### Issues related to participant recruitment

The eligibility criteria are carefully constructed to recruit non-professional athletes with serious knee injury who wish to return to physically demanding sports, to maximise the external validity of the trial. BANG trial recruitment centres perform at least one-third of all ACL reconstructions performed annually in Sweden [12]. Patients are recruited at the point of care, which means we recruit people in the usual care environment who present to the orthopaedic clinic, have the condition of interest, and have presented on their own behalf without any overt effort to recruit the person [45].

Because our inclusion criteria are stringent, we anticipate recruitment could be slow. Over half of all ACL reconstructions performed annually in Sweden are in patients aged between 16 and 30 years. The treatment algorithm in Sweden is for ACL reconstruction surgery to be recommended to young patients who wish to return to pivoting sports [46]. The Data Management Committee (DMC) will have access to participant recruitment information (including recruitment rates at each clinical site). It is within the DMC’s charter to review recruitment and make recommendations to modify recruitment should the DMC deem it necessary.

### Issues related to participant blinding

Maintaining participant blinding to the intervention is difficult in this trial. We have approached this challenge in two ways to try to minimise the risk of detection bias: (1) the smartphone application platform is used to deliver the Back in the Game app (Internet-delivered intervention) *and* for outcome measurement – the platform home screen and functions look the same to all participants, and (2) we inform participants that depending on their group allocation, they will receive the same amount of content as usual for people with ACL reconstruction or more content than usual for people with ACL reconstruction. Despite these approaches, we cannot assume that all participants will remain blind to group allocation for the duration of the trial.

### Issues related to pragmatic trial design

The BANG trial employs a pragmatic design that reflects the reality of and can inform clinical practice [47]. All participants will receive usual rehabilitation care following their ACL reconstruction – the Internet-delivered intervention is delivered *in addition* to usual rehabilitation care. Rehabilitation after ACL reconstruction is based on well-established clinical practice guidelines, we expect the goals of rehabilitation to be based on similar principles and structured in similar ways, and the outcomes of the rehabilitation provided to be relatively consistent across the recruitment sites.

Rehabilitation following ACL reconstruction typically continues for at least 6 months, and is tailored to the individual’s sports participation goals and function requirements. Because of the length of rehabilitation required before the athlete is physically and mentally ready to return to sport, and the requirement for tailoring, it is unreasonable to prescribe a standard rehabilitation protocol. A standardised rehabilitation programme would also require substantial resources to implement and render the trial infeasible.

### Issues related to adverse events

The most serious short-term negative consequence after ACL reconstruction and return to sport, is another serious knee injury (ligament rupture or meniscus tear). Sports exposure is the strongest risk factor for new knee injury; 1 in 5 young athletes who returned to sports after ACL reconstruction sustained a second ACL injury [48]. Given the Back in the Game app is designed to improve the return to sport rate after ACL reconstruction – a key patient-important outcome after surgery – there is a possible risk of paradoxical harm. It is within the DMC’s purview to examine adverse events, including new knee injuries, and make recommendations to protect the safety of participants.

## Supplementary information

**Additional file 1.**

## Data Availability

Not applicable, no datasets are included in this study protocol.

## Abbreviations

ACL: Anterior cruciate ligament
BANG: BAck iN the Game
TIDieR: Template for Intervention Description and Replication
ACL-RSI: ACL-Return to Sport after Injury
IKDC: International Knee Documentation Committee
K-SES: Knee Self-Efficacy Scale
ACL-QoL: ACL-Quality of Life
SANE: Single Assessment Numeric Evaluation
SMS: Short message service
DMC: Data monitoring committee
