# Supplementary material for: BAck iN the Game (BANG) – a smartphone application to help athletes return to sport following anterior cruciate ligament reconstruction: protocol for a multi-centre, randomised controlled trial
Source: BMC Musculoskelet Disord. 2020 Aug 8;21:523. doi: 10.1186/s12891-020-03508-7 (PMC7414541; doi:10.1186/s12891-020-03508-7)
Supplement: Supplementary file 1 — Additional file 1. [file 12891_2020_3508_MOESM1_ESM.pdf]

BAck iN the Game Trial: BASELINE CHARACTERISTICS

Do you smoke?
☐No
☐Yes (how many cigarettes per day)

Do you use snuff?
☐No
☐Yes (how many doses per week)

What is your main occupation?
☐Student
☐Employed
☐Searching for work
☐Other

What were you participating in when you injured your knee?
☐Organised sport
☐Work/employment
☐Recreation
☐Other

Do you have any other injury or illness that might affect your treatment after your knee surgery?
☐No ☐Yes (please describe the injury/illness and any treatment)

Back iN the Game Trial: RETURN TO SPORT

Return to sport goals

Have you returned to your desired sport or recreation activity?

- ☐ Yes
- ☐ No
    - For what reason have you not returned to your desired sport or recreation activity?
        - ☐ I am doing knee rehabilitation
        - ☐ I have poor knee function
        - ☐ I don't trust my knee
        - ☐ I'm scared about injuring my knee again
        - ☐ Another reason

Do you plan to return to the same sport as before your knee injury?

- ☐ I have returned to the same sport as before my injury
- ☐ I have returned to a different sport
- ☐ I plan to return to the same sport as before my injury
    - When do you think you will be back playing your sport?
        - ☐ Within one week
        - ☐ Within one month
        - ☐ Within six months
        - ☐ Within one year
        - ☐ Within more than one year
        - ☐ Another timeframe
- ☐ I do not plan to return to the same sport as before my injury
    - Why don't you aim to return?
        - ☐ I don't think my knee will be able to handle it
        - ☐ I don't want to risk getting injured again
        - ☐ I have lost interest in the sport I played before my knee injury
- ☐ I do not plan to return to sport

Motivation to return to sport

How important is it for you to return to your previous sports participation?

Not important	1	2	3	4	5	6	7	8	9	10	Very important

Do you think it is possible for you to return to your previous sports participation?

Impossible	1	2	3	4	5	6	7	8	9	10	Very possible

Back iN the Game Trial: RETURN TO SPORT

How willing are you to return to your previous sports participation?

Not willing	1	2	3	4	5	6	7	8	9	10	Very willing

BAck iN the Game Trial: PRIMARY OUTCOME

Question 1: Do you plan to return to the same sport as before your knee injury?
Response options:

(a) I have returned to the same sport as before my injury
(b) I have returned to a different sport
(c) I plan to return to the same sport as before my injury
(d) I do not plan to return to the same sport as before my injury
(e) I do not plan to return to sport

Question 2 (for participants who choose answer option *a* or *b* for Question 1)
Choose the most appropriate option
Response options

(a) I have returned to modified training
(b) I have returned to full training
(c) I have returned to full training and modified competition
(d) I have returned to full training and full competition

Question 3 (for participants who choose answer option *a* or *b* for Question 1)
Which sport?
Response options – select one of: group training/gym classes, basketball, cycling/spinning, football, dancing, handball, floorball, ice hockey, martial arts, running, motor sport, walking, swimming, cross-country skiing, alpine skiing, weights/strength training, tennis/squash, competitive team gymnastics, volleyball, other (specify).

Question 4 (for participants who choose answer option *a* or *b* for Question 1)
Which level?
Response options

(a) Elite
(b) Competitive
(c) Recreational

Back iN the Game Trial: SPORTS & PHYSICAL ACTIVITY PARTICIPATION

On a scale from 0 to 100, where 100 represents the best, which number would you give your knee today? _______________

How much time in total have you spent during the last 2 weeks doing physical exercise that makes you feel short of breath (e.g. running, gym, ball sports?

☐ 0 minutes
☐ Less than 60 minutes
☐ 1-2 hours
☐ 2-3 hours
☐ 3-4 hours
☐ More than 4 hours

Which activity were you most active in during the last 2 weeks? If you were active in another activity, you can provide detailed information in a separate question.

☐ Aerobics
☐ Basketball
☐ Cycling/spinning
☐ Football
☐ Dancing
☐ Handball
☐ Floorball
☐ Ice hockey
☐ Martial arts
☐ Running
☐ Motor sport
☐ Walking
☐ Swimming
☐ Cross country skiing
☐ Alpine skiing
☐ Strength/weights training
☐ Tennis/squash
☐ Competition gymnastics
☐ Volleyball
☐ Other (please specify)

What level did you train or compete at during the last 2 weeks?

☐ Elite
☐ Subelite competitive
☐ Recreational

How many times in the last 2 weeks did you participate in the activity? _________ times

How many minutes in total (regular training and competition) did you participate in the activity? ____________ minutes

Back iN the Game Trial: NEW KNEE INJURY REGISTRATION

Have you sustained an injury to either knee since you last answered a questionnaire?
☐No
☐Yes

Which knee?
☐Right ☐Left

What happened when you injured your knee? (free text description)

Have you sought care for your new knee injury?
☐No (why not? – free text description)
☐Yes (describe what sort of care – free text description)

Back iN the Game Trial: NEW KNEE INJURY REGISTRATION

BANG Trial ID: _________

Template completed by ___________________________ on ______________________ [date]

Information about new knee injury

Injury date:___________________________________

Injured knee: ____left ____right

Description of context of new knee injury occurrence (e.g. what was the person doing at the time, how did the injury happen)

Specific details about ACL injury mechanism (if applicable)

For contact injuries note whether contact was to injured knee, to other body part, and whether the contact was from another player or an object. For non-contact injuries note whether the person was landing from a jump, pivoting/cutting, decelerating.

Investigations and/or treatment (note which hospital/clinic/clinician if known)

Additional comments

BAck iN the Game Trial: CLINICAL ASSESSMENT FORM

Participant code:_____________ Assessment date:_________________________

Assessor name:___________________________

Passive knee range of motion

Left knee:____________degrees Right knee:___________degrees

Knee effusion (mark one box only)

- ☐ 0 (no fluid wave on downstroke)
- ☐ Trace (small fluid wave on medial knee with downstroke)
- ☐ 1+ (large fluid wave on medial knee with downstroke)
- ☐ 2+ (fluid wave spontaneously returns to medial knee after upstroke)
- ☐ 3+ (excess fluid that cannot be moved away from the medial knee)

Strength tests

	**Peak torque at 60°/sec (best of 5 repetitions)**	
	Left	Right
**Quadriceps**	__________Nm	__________Nm
**Hamstrings**	__________Nm	__________Nm

	**Peak torque at 180°/second (best of 15 repetitions)**	
	Left	Right
**Quadriceps**	__________Nm	__________Nm
**Hamstrings**	__________Nm	__________Nm

Hop tests

	Left	Right
**Single hop trial 1**	________m	________m
**Single hop trial 2**	________m	________m
**Triple hop trial 1**	________m	________m
**Triple hop trial 2**	________m	________m
**Side hop**	________hops	________hops

BAck iN the Game Trial: CLINICAL ASSESSMENT FORM

To measure knee range of motion with goniometer

Extension. The person is lying on her/his back. Rest the heel of the limb to be measured on a 15cm box so that the knee hangs freely. Measure the maximum extension and repeat on the other knee.

Flexion. The person is seated with legs extended. The person bends her/his knee as much as possible, using arms to help flex. Measure the maximum flexion and repeat on the other knee.

To measure knee effusion (stroke test)

The person is seated with legs extended. With 4 fingers, on the medial joint line, make 2-3 strokes upward and 1 stroke downward on the lateral side.

Complete strength and hop tests after an 8-minute warm-up (stationary bicycle or treadmill run at moderate exertion (12-16 – "somewhat hard" on Borg scale).

Strength tests

The starting position is approximately 110° knee flexion. The instruction is to "bend and straighten your knee as hard and as fast as possible". At 60°/second, complete 5 repetitions. At 180°/second, complete 15 repetitions. One repetition is a cycle from full (110°) knee flexion to as close to full extension as possible, to full flexion).

Hop tests (clasp hands behind back; always test the uninjured limb before the injured limb)

Single hop for distance. Stand on the test leg behind the start line. Hop as far forward as possible, taking off and landing on the same foot with a controlled, balanced landing. On landing, the foot must remain in the same place – no additional hops allowed – for up to 3 seconds. Measure the distance from the start line to the heel. Record the longest distance hopped for both limbs.

Triple hop for distance. Stand on the test leg behind the start line. Take 3 hops as far forward as possible, taking off and landing on the same foot with a controlled, balanced landing. On landing, the foot must remain in the same place – no additional hops allowed – for up to 3 seconds. No stoping allowed between hops. Measure the distance from the start line to the heel. Record the longest distance hopped for both limbs.

Side hop. Place two parallel strips of tape 40 centimetres apart on the floor. For the left leg, the starting position is standing to the right side of the parallel strips of tape. For the right leg, the starting position is standing to the left side of the parallel strips of tape. In 30 seconds, hop as many times as possible from side-to-side over the parallel strips of tape. Record the number of valid hops (when the foot does not touch the tape) for each leg.

BAck iN the Game Trial: DATA MONITORING COMMITTEE CHARTER

**Introduction**	
Trial name	BAck iN the Game (BANG) Trial<br>(NCT03959215)
Trial objectives	To test whether a custom smartphone application delivering cognitive-behavioural therapy to address confidence for returning to sport (Back in the Game app), is effective for improving the number of people who return to their preinjury sport and level following anterior cruciate ligament reconstruction.
Scope of this charter	The purpose of this document is to describe the roles and responsibilities of the DMC for the BANG trial, including the timing of meetings, methods of providing information to and from the DMC, frequency and format of meetings, statistical issues and relationship with other committees.

**Roles and responsibilities**	
Broad statement of aims of the committee	To protect and serve BANG trial participants, and to assist and advise the principal investigators of the BANG trial so as to protect the validity of the trial.
Terms of reference	The DMC should (i) receive and review the progress and accruing data of the BANG trial (consider data about safety and adverse events, recruitment rates, and the accuracy and completeness of data collection including missing data and rates of loss to follow-up), and (ii) provide advice on the conduct of the trial to the principal investigators. The DMC should inform the principal investigators if, in their view, one trial arm is clearly causing harm.
Specific roles of the DMC	• Monitor evidence of treatment harm (e.g. new knee injury)<br>• Decide whether to recommend that the trial continues to recruit participants or whether recruitment should be terminated either for all patients or some participant subgroups<br>• Suggest additional data analyses<br>• Consider the ethical implications of any recommendations made by the DMC<br>• Assess the impact and relevance of external evidence

**Composition**	
Membership and size of the DMC	The DMC will comprise at least 3 independent committee members (including at least one clinician and one researcher). One or more of the principal investigators may attend DMC meeting(s), but the decision-making is limited to the DMC members.
DMC Chair	The Chair should have experience of chairing meetings, and should be able to facilitate and summarise discussions. Ideally, the Chair will have previous experience of serving on a DMC. However, we will not make this mandatory for the BANG Trial DMC.<br>The DMC Chair will be chosen by consensus discussion between the principal investigators and DMC members.
Responsibilities of the statistician	The trial statistician will produce analyses for the DMC report, and will participate in DMC meetings. The trial statistician will guide the DMC through each report, participate in DMC discussion and, on some occasions, take notes.
Responsibilities of the co-ordinating principal investigator	The trial co-ordinator may help the trial statistician to produce the non-confidential sections of the DMC report. The trial co-ordinator will attend the open sessions of the DMC meetings, but not contribute to DMC decision-making.
Responsibilities of the trial investigators	The trial investigators may attend the open sessions of the DMC meetings (only if required), but may not contribute to DMC decision-making.

**Relationships**	
Clarification of the DMC role	The principal investigators will be responsible for trial co-ordination, supervision and administration.<br>An interim analysis of adverse events may be performed when 50% of participants have been randomised and have completed the 12-month follow-up. The interim results will be reported to the DMC, which will discuss the results and recommend whether the trial should continue. The DMC role is to advise the trial investigators.
Payment	DMC members will be reimbursed for reasonable travel and accommodation expenses where travel is required to attend in-person meetings.
Declaration of competing interests	DMC members will declare in writing any competing interests (real and potential) before the first DMC meeting. Completed competing interests forms will be returned to the co-ordinating principal investigator. Competing interests are not limited to financial interests, and may include involvement in other trials or intellectual investment.

Organisation of DMC meetings	
Meeting frequency	The frequency of meetings will be determined by the DMC Chair. We anticipate there might be one interim meeting and one final meeting. We plan for the DMC to have its first meeting within one year of recruitment commencing to discuss the trial, analysis plan and future meetings, and clarify roles and responsibilities of the DMC.
Meeting mode	We will endeavour to hold videoconferences wherever possible to reduce the time burden on DMC members.
Organisation of open and closed meeting sessions	The DMC Chair has the final say on meeting organisation. We plan for the following meeting format:<br>1. Open session: introduction and any ”open” parts of the report<br>2. Closed session: DMC discussion of ”closed” parts of the report<br>3. Open session: discussion with the co-ordinating principal investigator and any other relevant attendees on any matters arising from the preceding closed session.<br>4. Closed session: additional closed discussion, if required

Documentation, and procedures to ensure confidentiality and proper communication	
Material to be available in DMC meetings	*Open sessions*: accumulating information related to recruitment and data quality (e.g. recruitment rates, survey completion rates), total number of events for the primary outcome and summary measures for secondary outcomes may be presented, at the discretion of the DMC.<br>*Closed sessions*: in addition to the material available in the open sessions, the closed session material will include adverse events and reinjury data by treatment group.<br>The DMC members will not be blinded to treatment allocation.<br>Reports to the DMC will be available at least 1 week before any meetings.
The people who will see the accumulating data and interim analysis	The confidential accumulating data and interim analysis by treatment allocation will be seen by the DMC members and statistician.<br>DMC members do not have the right to share confidential information with anyone outside the DMC, including the principal investigators.
Responsibility for identifying and circulating external evidence	The co-ordinating principal investigator and other members of the BANG trial group will be responsible for collecting and circulating external evidence (e.g. from other trials or systematic reviews)

To whom the DMC will communicate their recommendations	The DMC will report its recommendation(s) in writing to the co-ordinating principal investigator.
What will happen to any confidential papers after DMC meetings	The DMC members should store confidential papers safely after each meeting so they may check the next report against them. After the trial is reported, the DMC members should destroy all interim reports.

**Decision-making**	
What recommendation(s) will be open to the DMC	Possible recommendations could include:<br>• No action needed; trial continues as planned<br>• Early stopping due to clear harm of treatment<br>• Extending recruitment or extending follow-up<br>An interim analysis of adverse events (using generalised estimating equations) may be performed when 50% of participants have been randomised and have completed the 12-month follow-up.<br><br>The DMC will review and agree any interim analysis plan.<br><br>The minimum dataset required for review will be:<br>• All adverse events<br>• All return to sport data
How recommendations will be reached within the DMC	Consensus discussion will be used to reach a final recommendation. The DMC Chair should summarise discussions and encourage consensus, and provide his/her opinion last. The Chair’s vote will be the deciding vote if the DMC cannot reach a unanimous decision. The DMC Chair will ensure the implications (e.g. ethical, statistical, practical, financial) for the trial are considered before any recommendation is made.
When the DMC is quorate for decision-making	All members should attend DMC meetings. The co-ordinating principal investigator will ensure a date and time is chosen to enable all members to attend DMC meetings. Meetings will be held via teleconference (with the possible exception of the first DMC meeting, if all members agree). If, at short notice, any member of the DMC cannot attend, the co-ordinating principal investigator will make arrangements for a new meeting.<br>If a DMC member cannot attend a meeting, it should be ensured that the member is available for the next meeting. If a member does not attend a second meeting, they should be asked if they wish to remain part of the DMC.

Reporting	
To whom will the DMC report their recommendations, and in what format?	The DMC recommendations will be summarised by the Chair and sent electronically to the co-ordinating principal investigator no later than 3 weeks following the DMC meeting.
DMC meeting minutes	The co-ordinating principal investigator will record minutes of the open sessions of DMC meetings. Minutes will be finalised by signature of the DMC Chair and stored by the co-ordinating principal investigator. Minutes of the closed sessions will be recorded by a DMC designee, finalised by signature of the DMC Chair, and stored securely by the DMC Chair.
Disagreement between the DMC and BANG trial principal investigators.	If the DMC has serious problems or concerns with the decisions of the principal investigators, a meeting of these groups should be held. The information to be shown would depend on the action proposed and the DMC's concerns. Depending on the reason for the disagreement, confidential data may have to be revealed to all attending such a meeting. The meeting should be chaired by an external expert who is not directly involved with the trial.

After the trial	
Publication of results	The DMC may wish to see a statement that the trial results will be published in a correct and timely manner.
Information about the DMC that will be included in published trial reports	DMC members will be named and their affiliations listed in the main trial report, unless they explicitly request otherwise.

BAck iN the Game Trial: CONSENT FORM

Linköping University, Institution for Medicine and Health in collaboration with Linköping Orthopaedic Clinic, Capio Artro Clinic, Stockholm, Capio Lundby, Gothenburg is conducting a study about returning to sport after surgery to the anterior cruciate ligament (ACL). The aim of the study is to investigate whether using the Back in the Game smartphone application can facilitate returning to sport after ACL surgery.

The app has questions and other material to support you in returning to your sport. If you accept to participate in the study, you will ether receive a little bit of material, or a lot of material. You will be given instructions about how to download the app, and log in details to access the material. You will then receive push notifications about once per week for six months with instructions about what to do.

You will also receive some questions about participating in sport and physical activity for two years after surgery. We will examine your knee function with, among other things, tests for muscle strength, at 1 year after your knee surgery.

Once we have collected the data, the results will be summarised and you will receive a short report of the main study results. The report will not contain individual results.

Participating in the study is voluntary, and you can stop participating whenever you wish without having to give a reason. Participating in the study does not affect your treatment in any way.

All people working on this research project have a responsibility to maintain confidentiality of all individual data. All data we collect are coded. All data in the app are encrypted and stored on Linköping University's server. Data are handled in accordance with the General Data Protection Regulations, GDPR (EU 2016/679), and you have the right to know what information is collected about you, request correction if there are errors, or request restriction/deletion of data.

The Data Protection Ombudsman can be reached at dataskyddsombud@regionostergotland.se. If you wish to file a complaint regarding the processing of your personal data, you can contact the Data Inspectorate, which is the supervising authority responsible for data protection. The p

Collected material may be used, in de-identified form, in other studies that have received ethical approval.

The study is approved by the Regional Ethics Review Board in Linköping. If you have questions or concerns during the study period, you can contact Professor Joanna Kvist, physiotherapist, Linköping University, joanna.kvist@liu.se, 013 284-664.

I accept to participate in this research study on returning to sport after ACL surgery. I have received verbal and written information about the study, and I understand that information. Any questions I had about the study have been answered.

Date:
Place:
Name:
Social security number:
Signature:
